# Statistical Conditional Sampling for Variable-Resolution Video Compression

**DOI:** 10.1371/journal.pone.0045002

**Published:** 2012-10-08

**Authors:** Alexander Wong, Mohammad Javad Shafiee, Zohreh Azimifar

**Affiliations:** 1 Department of Systems Design Engineering, University of Waterloo, Waterloo, Canada; 2 School of Electrical and Computer Engineering, Shiraz University, Shiraz, Iran; National Microelectronics Center, Spain

## Abstract

In this study, we investigate a variable-resolution approach to video compression based on Conditional Random Field and statistical conditional sampling in order to further improve compression rate while maintaining high-quality video. In the proposed approach, representative key-frames within a video shot are identified and stored at full resolution. The remaining frames within the video shot are stored and compressed at a reduced resolution. At the decompression stage, a region-based dictionary is constructed from the key-frames and used to restore the reduced resolution frames to the original resolution via statistical conditional sampling. The sampling approach is based on the conditional probability of the CRF modeling by use of the constructed dictionary. Experimental results show that the proposed variable-resolution approach via statistical conditional sampling has potential for improving compression rates when compared to compressing the video at full resolution, while achieving higher video quality when compared to compressing the video at reduced resolution.

## Introduction

Over the last two decades, digital video compression has become one of the fastest growing areas of research and development around the world, where the underlying goal is to take digital video content and encode it in a form that minimizes the requirements for digital storage and/or transmission. There is a continually increasing demand for better digital video compression technologies, particularly since digital video has become an integral part of our daily lives, with mass digital video consumption in a wide range of application scenarios such as digital TV broadcast (via MPEG-2 [1] in most North American systems), real-time Internet video streaming, real-time video telecommunications (e.g., via H.32x [2]), personal video recording, and media disk storage (e.g., DVDs). Given the incredible demand for high quality digital video content consumption, significant progress has been made in the area of digital video compression, cumulating in the current state-of-the-art video compression standards such as H.264/MPEG-4 AVC [3], a block-transform motion-compensated based digital video codec standard that provides significantly improved compression rates when compared to previous standards. Much of the gains in compression performance over the past two decades in digital video compression has been largely due to improvements on rate-distortion optimization techniques [4]–[7] and motion compensation techniques [8]–[12] such as improved inter-frame utilization, variable block size motion compensation, multiple motion vectors per macroblock, and sub-pixel motion compensation precision.

Despite the great increases in compression performance gained through rate-distortion optimization and motion compensation, another area of research in digital video compression that has garnered recent interest and is worth investigating is the area of variable resolution compression [13]–[17], where the underlying video content is stored and compressed at different spatial resolutions. In the work by Wei et al. [13], salient regions are detected within the scene via a visual attention model. The regions with the highest saliency is stored and compressed at its original resolution, the regions with lowest saliency stored at medium resolution, and regions in between stored at medium resolution. In the work by Defroges et al. [15]–[17], referred to as locally adaptive resolution (LAR) techniques, regions of interest are extracted from the scene via a region segmentation approach. These regions of interest are then reduced in resolution depending on the underlying content, such that smaller regions maintain higher resolution for the underlying video content while larger regions are stored and compressed at a reduced resolution.

One of the main limitations with existing variable resolution compression techniques is that they are largely constrained to exploiting spatial redundancy within a video frame. As such, the significant information redundancy that can be gained by considering the spatial-temporal characteristics of the underlying video content is largely untapped in current methods. Furthermore, existing variable resolution methods require significant modifications and even architectural departures from current state-of-the-art video compression standards. As such, a method that addresses both issues is worth investigating.

The main contribution of this paper is to introduce and investigate the potential for the use of Conditional Random Fields and statistical conditional sampling for variable-resolution video compression, with the aim to improve compression rates while maintain high visual quality. Rather than store individual regions within a frame at different resolutions, as previous approaches have done, we take a radically different approach where different frames within a video are stored and compressed at different resolutions. At encoding, the keyframes are stored at full resolution, while the rest of the frames are stored at reduced resolutions. At decoding, a region-based dictionary from high resolution representative key-frames within a video shot is constructed automatically and statistical conditional sampling based on Conditional Random Field is used to restore the low resolution frames to the original resolution based on information contained within the full resolution dictionary of regions. By incorporating the proposed approach within a H.264 video compression framework, the proposed approach can take advantage of all the advanced rate distortion optimization and motion compensation techniques inherent and available for H.264 while provided an additional value-added component for improving compression performance over the existing framework.

The rest of the paper is organized as follows. First, the underlying methodology behind the proposed use of Conditional Random Fields and statistical conditional sampling for variable-resolution video compression is described in Section. The experimental results and the associated discussion is presented in Section. Finally, conclusion are drawn and future work is discussed in Section.

## Methods

**Figure 1:**
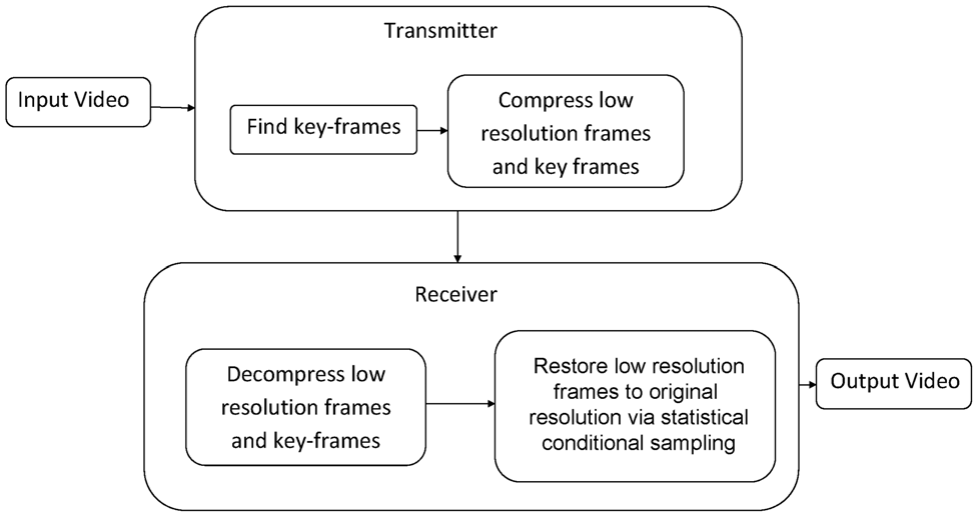
Flow diagram of the proposed variable-resolution approach.

The proposed use of statistical conditional sampling for variable-resolution video compression consists of two main stages: i) Variable-resolution compression stage, and ii) Decompression stage. In the variable-resolution compression stage, the identified representative key-frames are compressed at full resolution while the rest of the frames are compressed at a lower resolution. Secondly, in the decompression stage, all of the frames within the video content are decompressed at their respective resolutions, a full resolution region-based dictionary is constructed from the representative key-frames and then the low resolution frames are restored to the original resolution via statistical conditional sampling based on the dictionary and conditional probability of CRF. An flowchart summarizing the proposed approach is shown in Fig. 1.

### Representative frame identification stage

At the first step in compression stage, representative key-frames are identified and extracted within a video shot. To achieve this goal, we wish to first determine an appropriate metric for quantifying the similarity between every frame pair within the shot. Given the importance of structures of objects within a scene, we chose to employ the well-known SSIM metric proposed by Wang et al. [18], which has been shown to provide a strong indicator for visual similarity assessment in a local manner. The local nature of the metric is important since:

- video statistical features are usually highly spatially/temporally nonstationary,
- video distortions may also be space/time variant,
- at one time instance and at a typical viewing distance, only a local area in the image can be perceived with high resolution by the human observer, and
- a localized quality measurement can provide a space-time varying quality map of the image, which delivers more information about the quality degradation of the image and may be useful in some applications.

Having considered the above properties, the SSIM metric can be defined as
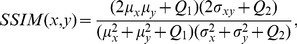
(1)where the constant 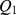 is included to avoid instability when 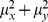 is very close to zero. Specifically, 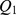 is chosen as the squared product of pixel values dynamic range and a small positive constant much less then one. Similarly, the constant 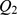 is assumed as the squared product of pixel values dynamic range and another small constant.

To utilize the SSIM metric for assessing video frame similarity so that we can identify representative keyframes, an SSIM matrix (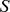) is first constructed, where the elements of the matrix indicate the SSIM value between every two frames within a shot. A distance matrix 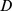 is then calculated to obtain the temporal distance map of the given shot,
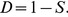
(2)For each frame 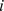 within the video shot, a vector with size 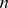 depicts the distance of the frame and other 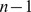 frames, where the 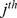 entry of the 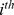 vector shows the SSIM of frame 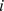 and frame 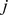.

In order to avoid identifying uniformly distributed key-frames, a Fuzzy c-means clustering strategy [19] is employed to identify the representative keyframes within the video shot. To determine the number of clusters (i.e., the actual number of representative keyframes to store), a principal component analysis (PCA) approach is utilized, where one can determine the significant eigenvalues within the set of data and use them to determine a reasonable estimate of the number of clusters, i.e., number of representative key-frames within the shot to store. This proposed key-frame identification and selection process is important since the number of keyframes that needs to be stored and compressed can vary greatly from shot to shot depending on the underlying video content.

**Figure 2:**
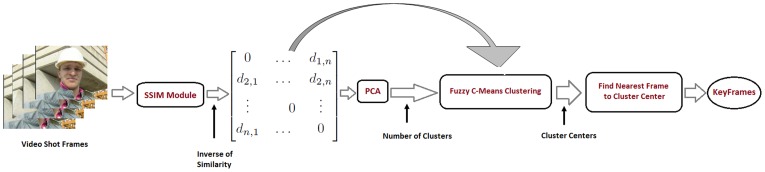
Flow diagram of the representative keyframe identification and selection procedure.

Based on the above theory, the representation keyframe identification and selection procedure from a video shot can be described in detail as follows (Fig. 2). Suppose that the video shot 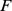 contains 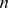 frames 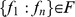. Because we wish to select the most informative and representative frames as the representation keyframes, the similarity of each pair of frames is calculated using the SSIM measure (defined in Eq. 1). The similarity between a reference frame 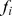 and a secondary frame 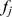 will be denoted as 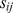. Based on the similarity 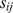, one can get an assessment of dissimilarity 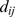 as its inverse: 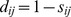. Therefore, the dissimilarity matrix 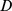 representing the dissimilarities between all frames in the video shot as:
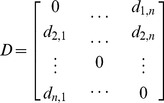
(3)where 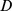 is sample space which will be utilized to identify the keyframes. Each row 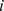 of 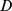 is the corresponding feature vector for frame 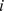. As mentioned, first of all, PCA is used to determined the sufficient number of keyframes to be identified for the video shot. Based on the covariance 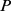 of the sample space 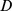, the number of keyframes 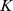 is specified to be the number of significant eigenvalues.

Once the number of clusters 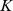 is determined, the fuzzy c-means (FCM) clustering procedure is used to select the most informative and representative keyframes. The FCM algorithm attempts to partition a finite collection of 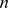 elements 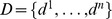 into a collection of 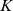 fuzzy clusters. Given a finite set of data, the algorithm returns a list of 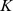 cluster centers 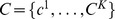 and a partition matrix 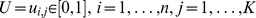 (Eq. (4)). Each element 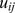 characterizes the degree to which element 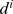 belongs to cluster 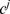:
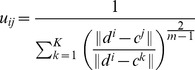
(4)

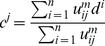
(5)This procedure is iterated 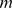 times until convergence is achieved. After the procedure converges, the 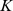 clusters are identified. To find the keyframes, the nearest sample 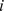 to each cluster center 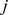 is determined based on minimum distance:
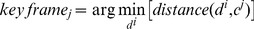
(6)At this stage, the representative keyframes have been selected and are stored at the original resolution.

### Variable-resolution compression stage

In the variable-resolution compression stage, the frames within the video content are stored and compressed via H.264 [3] depending on whether it is one of the identified representative key-frames or not. For the set of frames that are not identified as representative key-frames, they are down-scaled to a lower resolution and compressed as a video sequence at this reduced resolution. For implementation purposes, this set of frames are down-scaled by a factor of 2 in both the vertical and horizontal resolutions. The keyframes are compressed at the original resolution. By compressing them at the original resolution, much of the important details within the frames are well preserved, which is fundamental for the decompression stage when we attempt to restore the lower resolution video frames to their original resolutions. The main advantage of this compression approach is that a state-of-the-art video compression standard such as H.264 can be used directly for variable-rate video compression without the need for significant modifications, making it well suited for integration into consumer level media devices.

### Decompression stage

In this stage, the goal is to reconstruct the decompressed video content back to the original resolution. First, the representative key-frames are decoded and decompressed at full resolution, while the rest of the frames are decoded and decompressed at the reduced resolution. In this stage the region-based dictionary 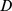 is constructed from full resolution key-frames. Once we have the decompressed frames, we restore the low resolution frames to the original resolution via statistical conditional sampling, which is described as follows.

Let 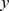 is a realization of low resolution frame 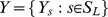, and 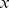 is a realization of original resolution frame 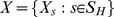, where 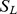 is the set of all pixels within the low resolution frame, while 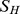 is the set of all pixels within the original resolution frame. The conditional probability of 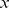 given 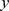 can be expressed as:
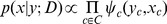
(7)where 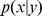 is modeled by Conditional Random Field (CRF) [20] (a parametric model) in which 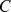 is the set of clique templates, 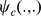 is a potential function, and 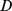 is the dictionary of high resolution regions which were extracted from key-frames. We can determine the original resolution frame 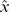 by sampling from 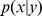:
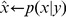
(8)while various potential functions 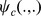 can be applied, the most simplest and effective one is Sum of Squared Difference (SSD). As the objective of this paper is to find the best high resolution frame based on the low resolution compressed video frame and the key-frames, SSD was found to be an appropriate metric.

#### Training

The only feature function utilizing in this paper is the SSD measure, therefore, the training phase of CRF simply is to determine the key-frames utilized to construct the dictionary 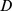 [21]. For efficient implementation purposes, the region-based dictionary 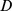 is constructed for each pixel 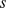 in the following manner. First, a total of 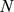 samples are randomly drawn from a 2-D Gaussian sampling distribution with a mean of 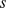 and a standard deviation of 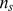. At the pixel locations corresponding to each of the 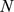 samples, a 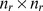 high resolution region around that pixel is extracted from the representative high resolution keyframes and stored into the dictionary. In this study, 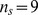 and 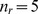 as they were found experimentally to provide strong visual quality.

#### Sampling and inference

**Figure 3:**
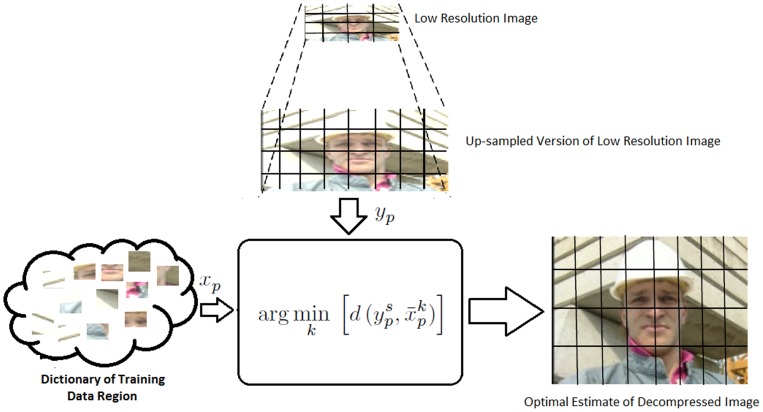
Flow diagram of the sampling and inference step.

The sampling is done to find the best matching high resolution frames. The original resolution frame 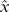 is estimated by directly sampling from the dictionary of region-based training data 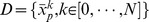 according to 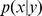. This is accomplished by computing the optimal estimate 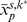 for reconstructing the original resolution frame by identifying the best regional match for each pixel 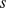 in an up-scaled version of the low resolution frame 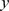, denoted as 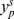, with the dictionary of regions 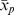,
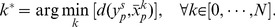
(9)where 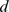 is the dissimilarity metric between two regions (for implementation purposes, 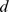 is the sum of squared differences between the regions), and the clique definition on this approach is based on the 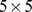 neighborhood structure. Once the best matching region from the dictionary 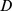 for a pixel 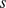 is determined, the value at 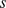 in the estimated original resolution frame 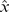 is set to the value of the center pixel of that best matching region. The overview workflow of the sampling and inference step is shown in Fig. 3.

## Results and Discussion

To demonstrate the potential of the proposed use of statistical conditional sampling for variable-rate video compression, a number of different video sequences were tested. Two main performance metrics were evaluated. First, we evaluate the compression rate achieved using the proposed method against the compression rate achieved by: 1) compressing the entire video sequences at full resolution using H.264 [3], and ii) compressing the entire video sequences at a reduced resolution of a factor of two for both horizontal and vertical resolutions using H.264 [3]. H.264 [3] is a state-of-the-art video compression framework that accounts for inter-frame redundancy. This performance metric allows us to evaluate whether the proposed variable-resolution approach's claims for improving compression performance is valid. Second, we evaluate the average peak signal-to-noise ratio (PSNR) and the average structural similarity index (SSIM) values of the video frames produced using the proposed approach, and compare to that achieved by compressing the entire video sequence at a reduced resolution. This performance metric allows us to evaluate whether the proposed approach's claims for improved video quality over compressing at a reduced resolution is valid.

Table 1 demonstrates compression ratio of the tested scenarios for each frame sequence. It can be observed that the compression ratios achieved using the proposed variable-resolution approach is noticeably higher than that achieved using the full resolution approach, which justifies the claim for the proposed approach of improving compression performance. When compared to the compression ratios achieved by the low resolution approach, the proposed approach takes a minor hit in storage overhead for the ‘Foreman’ and ‘Table Tennis’ video sequences, while taking a larger hit in storage overhead for the ‘Ohaio1’ and ‘Ohaio2’ video sequences. However, despite the storage overhead when compared to the low resolution approach, the overall compression performance of the proposed variable-resolution approach is still strong.

**Table 1:** The compression ratio of different sequences for the following scenarios: i) compression at full resolution (FR), ii) compression at low resolution (LR), and iii) compression via variable-resolution (VR) approach.

	Compression ratio		
Sequence	FR	LR	VR
Foreman	5.5∶1	9.31∶1	7.68∶1
Table Tennis	3.98∶1	14.84∶1	12.44∶1
Ohaio1	6.48∶1	20.90∶1	15.90∶1
Ohaio2	6.52∶1	26.26∶1	17.96∶1

**Figure 4:**
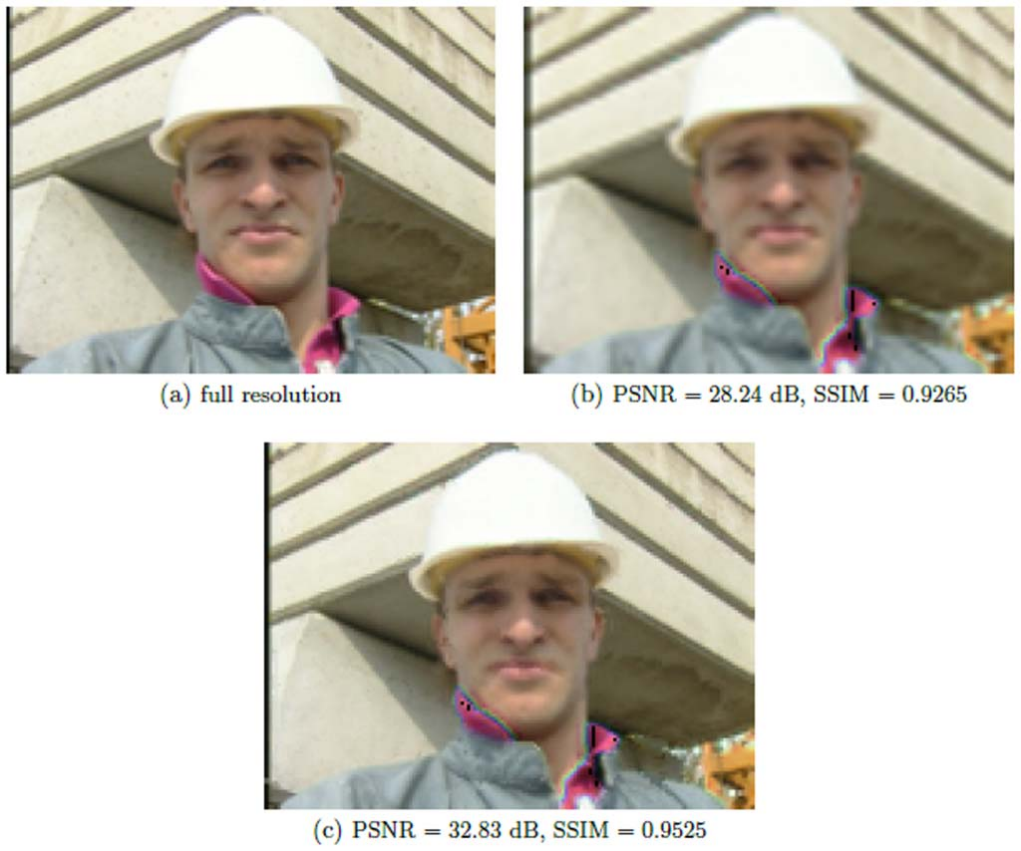
Visual comparison of the proposed method on two example frames from ‘Foreman’ video sequence [**22**]. (a) full resolution original frames, (b) Results of low resolution video compression and (c) depicts result of proposed variable-resolution compression approach.

**Figure 5:**
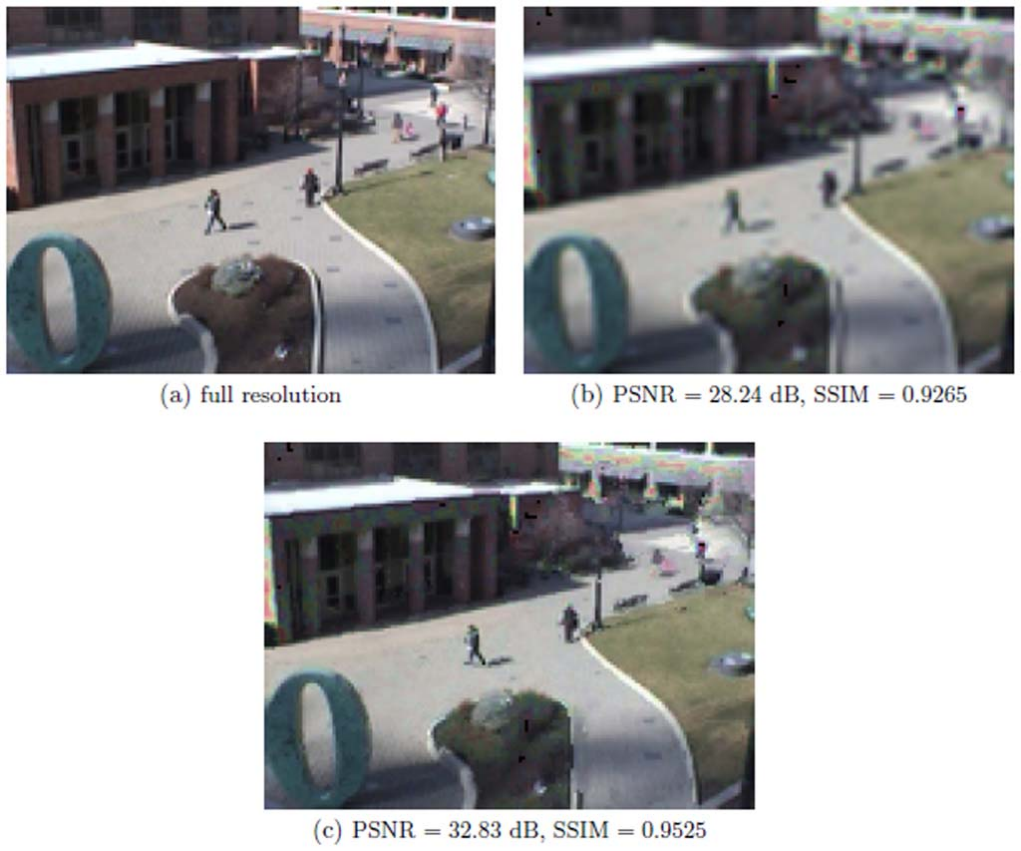
Visual comparison of the proposed method on two example frames from ‘Ohaio1’ video sequence. (a) full resolution original frames, (b) Results of low resolution video compression and (c) depicts result of proposed variable-resolution compression approach.

Table 2 shows the average PSNR and average SSIM values for the proposed use of statistical conditional sampling for variable-resolution video compression and for the scenario where the entire video sequence is compressed at a lower resolution. To facilitate for comparison purposes, the video frames from the low resolution scenario is up-scaled using bi-cubic interpolation so that it can be compared against the reference full resolution video frame. It can be observed that strong PSNR and SSIM values were obtained using the proposed approach for all video sequences when compared to the low resolution approach. Furthermore, a visual comparison of the proposed approach on two example frames from the ‘Foreman’ and ‘Ohaio1’ video sequences are shown in Fig. 4 and Fig. 5, respectively. It can be observed that the frames produced using the proposed approach contains noticeably more detail when compared to the frames produced using low resolution compression, thus validating the claim that improved visual quality can be achieved using the proposed approach. However, as expected, the visual quality of the frames produced using the proposed approach is not as good as the full resolution original frames, thus illustrating the trade off between visual quality and compression performance.

**Table 2:** Averaged PSNRs (dB) and SSIMs of the reconstructed frames for low resolution (LR) compression and the proposed variable-resolution (VR) compression approach.

	PSNR (dB)		SSIM	
Sequence	LR	VR	LR	VR
Foreman	27.77	31.27	0.9211	0.9448
Table Tennis	26.68	27.40	0.7280	0.7791
Ohaio1	25.29	26.90	0.8596	0.8811
Ohaio2	26.56	28.26	0.8450	0.8853

### Conclusions

The potential use of statistical conditional sampling for variable-resolution video compression to further improve compression rate while maintaining high quality video was studied. In the proposed approach, the representative key-frames were first identified within a video shot. The representative key-frames were compressed at the original resolution while the remaining frames within the video shot are compressed at a reduced resolution. Upon decompression, the reduced resolution frames are restored to the original resolution via statistical conditional sampling based on the original resolution representative keyframes. Experimental results demonstrate the potential of the proposed approach for improving compression rates when compared to compressing the video at full resolution, while achieving higher video quality when compared to compressing the video at reduced resolution. Future work involves exploring improved key-frame identification methods as well as improved frame restoration approaches.
